# Change in the Morphology of Alloy Corrosion Products Based on the FeAl Intermetallic Phase After Oxidation in Water Vapor at a Temperature of 700 °C for up to 2000 h

**DOI:** 10.3390/ma18225150

**Published:** 2025-11-12

**Authors:** Janusz Cebulski, Dorota Pasek, Maria Sozańska, Magdalena Popczyk, Jadwiga Gabor, Andrzej Swinarew, Jakub Wieczorek

**Affiliations:** 1 Department of Materials Technology, Faculty of Materials Engineering and Industrial Digitalization, Silesian University of Technology, Krasińskiego 8, 40-019 Katowice, Poland; maria.sozanska@polsl.pl (M.S.); jakub.wieczorek@polsl.pl (J.W.); 2 Promobil s.c., Kopernika 12, 40-064 Katowice, Poland; dorota.pasek@promobil.pl; 3 Faculty of Science and Technology, University of Silesia in Katowice, 75 Pułku Piechoty 1A, 41-500 Chorzów, Poland; 4 Institute of Sport Science, The Jerzy Kukuczka Academy of Physical Education, Mikołowska 72A, 40-065 Katowice, Poland

**Keywords:** α-Al_2_O_3_, intermetallics phase, oxidation layer

## Abstract

The surface of the Fe40Al5Cr0.2TiB alloy, after oxidation in steam at 700 °C, showed a varied morphology dependent on oxidation time. Initially, a fine, acicular oxide layer formed, which over time transformed into a more compact, lumpy structure corresponding to the α-Al_2_O_3_ phase. EDS analysis confirmed the dominance of aluminum and oxygen in the oxidation products, and XRD studies revealed the presence of the α-alumina phase. Optical profilometry revealed a significant increase in roughness parameters (R_a_ and R_z_) after long-term exposure (2000 h), which correlates with the thickening and sinterization of the oxide layer. The obtained results indicate that in a water vapor environment, a stable α-Al_2_O_3_ phase can already be formed at a temperature of 700 °C, and its development leads to increased roughness.

## 1. Introduction

The oxidation behavior of Fe–Al intermetallics (notably FeAl and related iron aluminides) is of fundamental and practical interest for high-temperature applications because these materials can form protective Al_2_O_3_ scales that markedly slow further oxidation [1,2,3]. The formation, continuity, and crystallographic form (polymorph) of alumina are critical: a continuous, adherent α-Al_2_O_3_ layer provides the best long-term protection, whereas transient or non-continuous alumina permits rapid inward/outward transport of species and can lead to breakaway oxidation [4,5,6,7].

Steam (H_2_O(g)) environments modify oxidation chemistry and transport in several important ways: they change the effective oxygen activity at the oxide/gas interface, enable hydroxyl-mediated transport pathways, and can promote volatile species and internal oxidation processes that are not active in dry O_2_ or air. Consequently, alloys and coatings that remain protective in dry oxygen may experience morphological changes, multi-layer scale formation, or accelerated degradation in steam atmospheres—effects that become increasingly important for energy systems operating in the 600–750 °C window (e.g., ultra-supercritical steam cycles, boilers, steam turbines, nuclear claddings). Experimental and review studies document that steam commonly produces outward Fe-rich nodules, inner (Fe, Cr) or spinel layers, and can increase spallation/scale porosity compared with dry exposures [8,9,10,11,12,13,14].

For Fe–Al systems, the literature shows a complex, composition- and microstructure-dependent response. Short-to-moderate exposures often produce continuous Al_2_O_3_ on aluminide coatings and some bulk FeAl alloys, yet long-term steam exposures may reveal: (a) development of multi-layered scales with Fe-rich outer oxides; (b) internal oxidation of Al beneath the surface; (c) Al depletion at the metal/scale interface due to interdiffusion; and (d) mechanical failure of the scale (cracking, spallation) driven by growth stresses and phase transformations [2,6,9,15,16,17,18]. Alloying elements (Cr, Si, Y, reactive elements) and microstructural features (grain size, second phases, porosity, pre-existing defects or scratches) strongly influence whether protective alumina persists or the system transitions to a non-protective morphology [6,12,19,20,21].

Despite the substantial body of work on steam oxidation of steels, Ni-base alloys, and aluminide coatings, there is a relative paucity of systematic, high-resolution, long-duration studies that document the morphological evolution of corrosion products specifically for bulk alloys whose matrix is the FeAl intermetallic phase exposed to steam at 700 °C for times approaching 2000 h. Many prior investigations focus on shorter times, on coated steels rather than bulk FeAl intermetallics, or on mixed O_2_/H_2_O atmospheres. Where long steam exposures have been reported, important morphologies such as nodule coalescence, multi-layer scale formation, and Al-depletion have been observed but not comprehensively correlated with high-resolution microstructural analyses across a wide time span (hours → thousands of hours) [3,9,10,11,13,22,23,24,25,26].

The present study fills this gap by performing a time-series analysis of oxide morphology and composition on a FeAl-based alloy exposed to H_2_O(g) at 700 °C for exposure times up to 2000 h. We combine surface SEM/EDS, where appropriate, XRD phase analysis, to classify oxide morphologies as a function of exposure time; identify transitions from protective alumina-dominated scales to complex multi-layered or Fe-rich morphologies; and assess the role of alloy chemistry and initial microstructure (including scratches/defects) in governing scale integrity and protectiveness.

These results provide mechanistic insight relevant to material selection and coating design for long-term service in steam environments, and supply a detailed dataset (morphology classes, composition profiles, and kinetic fingerprints) that can support modeling efforts and practical guidelines for Fe-Al-based materials and aluminide coatings in power-plant and related high-temperature steam applications.

## 2. Materials and Methods

The tests were conducted on samples made of the Fe40Al5Cr0.2TiB alloy, the chemical composition of which is presented in Table 1. ARMCO iron (technically pure), ARO aluminum (99.995 wt%), aluminothermic chromium obtained by the Kroll method, and amorphous boron (technically pure) were used for melting. Melts were carried out in a Balzers VSG-2 induction vacuum furnace (Balzers, Lichtenstein). Due to the fact that the as-cast material was brittle and had a coarse-grained structure and significant segregation of chemical composition, heat treatment was applied. Homogenizing annealing was carried out at 1050 °C for 72 h, as previous studies had shown that these are the parameters at which microsegregation of chemical composition is removed [27,28].

Corrosion tests at 700 °C in a flowing water vapor environment were performed on a test stand designed for isothermal oxidation corrosion. Oxidation times were 1, 50, 100, 300, 1000, and 2000 h. Oxidation in a steam environment was conducted in a furnace consisting of a tubular reactor divided into two zones. The first zone was the furnace’s corrosion chamber, while the second zone was the steam superheater. A steam generator was mounted on the front panel of the furnace, with a capacity of 200 mL/h during the test. During the test, steam was continuously supplied to the steam superheater and then to the corrosion chamber. Distilled water was used to generate the steam. Observations of the oxide layer surface of the Fe40Al5Cr0.2TiB intermetallic alloy samples after oxidation for initial oxidation times (1 h, 50 h, and 100 h) were performed using a ZEISS SUPRA 35 high-resolution scanning electron microscope (Carl Zeiss, Oberkochen, Germany). Observations of the surface state after oxidation for 300, 1000, and 2000 h were performed using a Hitachi S-3400N scanning electron microscope (SEM) (Hitachi High-Tech Corp., Tokyo, Japan) with secondary electron (SE) and backscattered electron (BSE) detectors, and a Hitachi S-4200 microscope with secondary electron (SE) detectors (Hitachi High-Tech Corp., Tokyo, Japan). The chemical composition of the oxidation products was determined using a Thermo Noran (System Six) energy-dispersive X-ray spectrometer (EDS) (Waltham, MA, USA) at an electron beam acceleration voltage of 15 keV, coupled to a Hitachi S-3400N microscope. Details of the scale morphology were also observed using a TESCAN S-8000 BrightBeam™ SEM at a voltage of 5 kV (TESCAN, Brno, Czech Republic). Surface development was measured using a non-contact optical profilometer with a MicroProf 3000 (FRT GmbH, Bergisch Gladbach, Germany) white light aberration head.

## 3. Results

### 3.1. Surface Morphology and Oxide Evolution (SEM/EDS/XRD)

**Figure 1:**
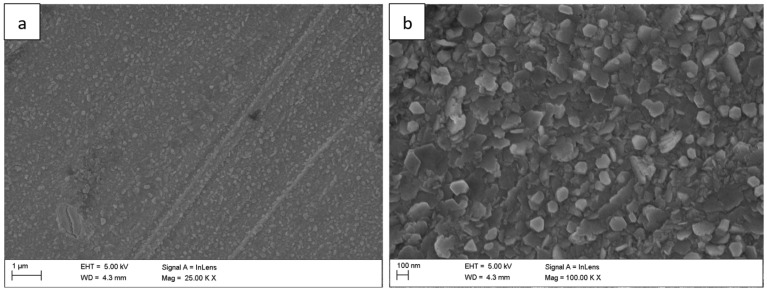
Surface morphology of the Fe40Al5Cr0.2TiB alloy after oxidation in water vapor at 700 °C for 1 h: (**a**) low-magnification SEM image showing general oxide coverage on the alloy surface; (**b**) high-magnification SEM image showing the morphology of fine, needle-like oxide.

**Figure 2:**
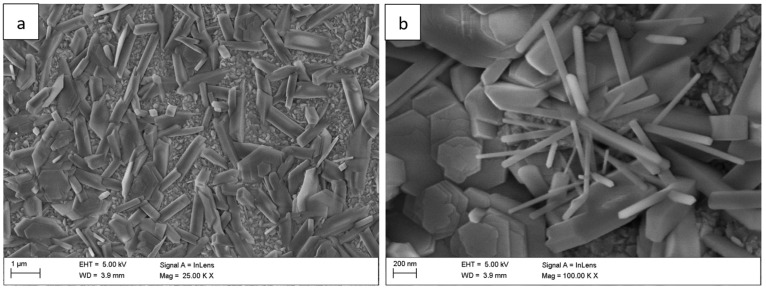
Surface morphology of the Fe40Al5Cr0.2TiB alloy after oxidation in water vapor at 700 °C for 50 h: (**a**) low-magnification SEM image illustrating the formation of a continuous oxide layer; (**b**) high-magnification SEM image showing partial coalescence and morphology of fine, needle-like oxide whiskers.

**Figure 3:**
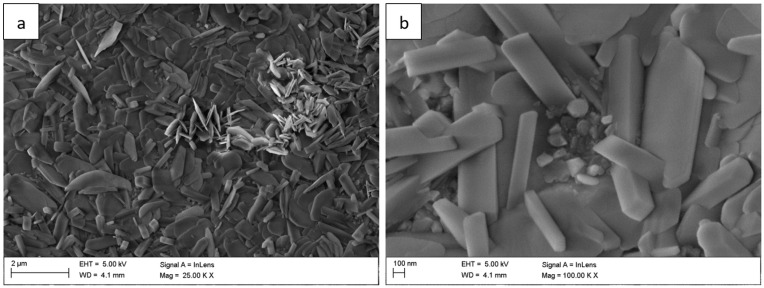
Surface morphology of the Fe40Al5Cr0.2TiB alloy after oxidation in water vapor at 700 °C for 100 h: (**a**) low-magnification SEM image showing the progressive densification of the oxide scale; (**b**) high-magnification image revealing clustered grains with partially flattened morphology.

The surface morphology of the Fe40Al5Cr0,2TiB alloy after oxidation in water vapor at 700 °C evolved significantly with exposure time. After 1 h (Figure 1), a relatively continuous, fine-grained oxide layer covered the surface, consisting mainly of needle-like structures (whiskers) and small agglomerates (flakes). These elongated oxides grew perpendicularly to the substrate, forming a columnar morphology typical of the metastable θ-Al_2_O_3_ polymorph [1,2,5]. As the exposure time increased to 50 h (Figure 2) and 100 h (Figure 3), whisker density decreased, and the oxide became denser, forming clustered grains with a partially flattened morphology.

**Figure 4:**
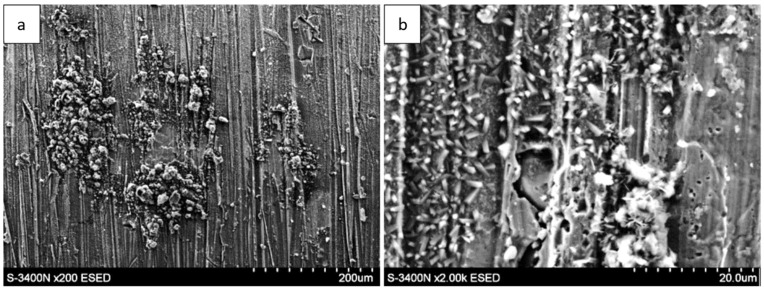
Surface morphology of the Fe40Al5Cr0.2TiB alloy after oxidation in water vapor at 700 °C for 300 h: (**a**) low-magnification image showing heterogeneous distribution of oxide clusters; (**b**) high-magnification image presenting the transition from whisker-like to compact granular structures.

**Figure 5:**
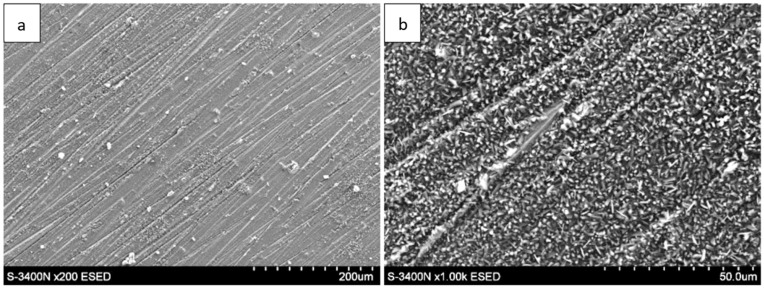
Surface morphology of the Fe40Al5Cr0.2TiB alloy after oxidation in water vapor at 700 °C for 1000 h: (**a**) low-magnification image of the surface showing growing granular regions; (**b**) high-magnification image showing morphology oxide.

**Figure 6:**
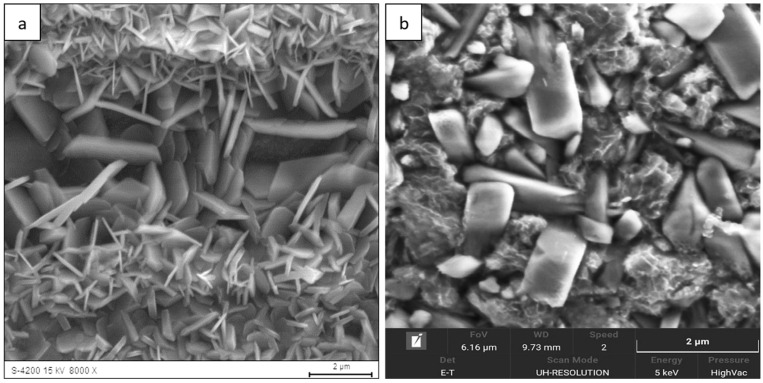
Surface morphology of the Fe40Al5Cr0.2TiB alloy after oxidation in water vapor at 700 °C for 2000 h: (**a**) low-magnification SEM image showing coarse granular agglomerates distributed over the surface; (**b**) high-magnification SEM image showing sintered α-Al_2_O_3_ nodules and locally compacted regions.

For long-term oxidation (≥1000 h, Figure 4 and Figure 5), the whisker-like features gradually transformed into compact, globular clusters distributed heterogeneously across the surface. After 2000 h (Figure 6), the oxide layer exhibited coarse granular agglomerates and locally sintered regions, indicating a transformation toward the stable α-Al_2_O_3_ structure [3,4,8]. The morphological transition from fine whiskers to dense nodules corresponds to the thermally activated θ → α phase transformation, which is associated with decreasing defect density and a reduction in internal energy per unit volume [5].

Similar morphological evolution has been reported for FeAl and FeCrAl alloys oxidized in dry air or steam at 750–950 °C [13,29]. Notably, the present results confirm that prolonged oxidation even at 700 °C in high H_2_O(g) activity promotes the nucleation and growth of α-Al_2_O_3_, likely due to local temperature gradients and Al diffusion from the substrate. This observation aligns with the findings of Cebulski et al. [6] and Grabke [1], who indicated that the presence of Cr and Ti additions facilitates alumina stabilization at lower temperatures by altering interfacial energy and vacancy concentrations.

**Figure 7:**
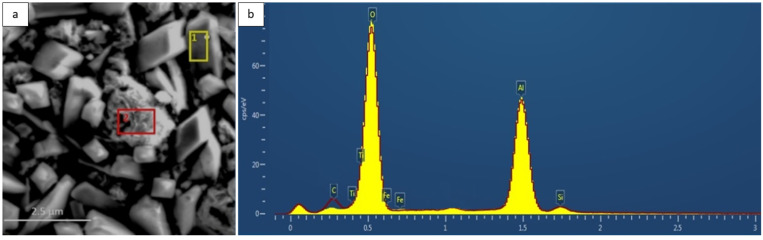
(**a**) Surface microstructure of the Fe40Al5Cr0.2TiB alloy after oxidation in water vapor at 700 °C for 2000 h; two EDS analysis points are indicated (red and yellow). (**b**) EDS spectra from the corresponding areas shown in (**a**): the red line represents the spectrum from the red-marked area, and the yellow line represents the spectrum from the yellow-marked area. The spectra overlap due to the very similar chemical composition of both regions (Al and O with minor Fe, Cr, and Ti).

EDS analysis revealed that the oxide layer contained predominantly aluminum and oxygen, with trace amounts of Fe, Cr, and Ti (Figure 7). The presence of these substrate elements in the outer scale results from the relatively deep excitation volume during EDS measurements and local diffusion processes at the metal/oxide interface [10,11]. In some areas, minor Si contamination was observed, originating from the furnace lining material.

**Figure 8:**
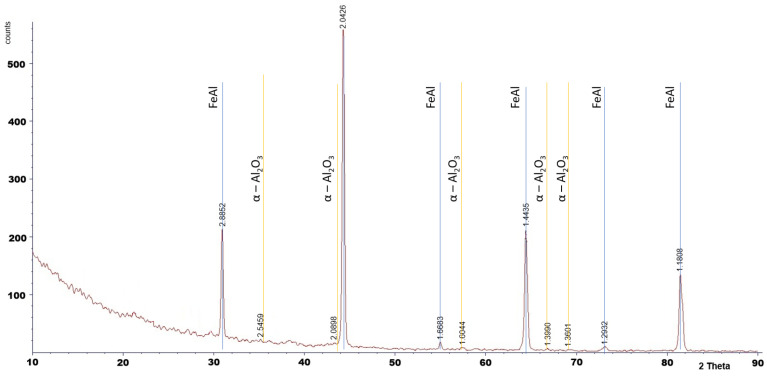
X-Ray analysis of the Fe40Al5Cr0.2TiB alloy after oxidation in water vapor at 700 °C for 2000 h.

Phase analysis by XRD confirmed the presence of α-Al_2_O_3_ after 2000 h of oxidation (Figure 8).

The XRD analysis was performed only for the specimen oxidized for 2000 h. For shorter oxidation times, the oxide layers were too thin to produce detectable diffraction peaks, as XRD is a bulk-sensitive technique. Phase identification for shorter exposures was therefore supported by SEM observations and literature reports.

The transition of whisker morphology (θ-Al_2_O_3_) into granular α-Al_2_O_3_ was also observed for NiAl alloys at 1000–1100 °C [5,30] and for FeCrAl coatings at 850–900 °C [8,16]. In this study, the same evolution occurred at 700 °C, suggesting that water vapor may accelerate the stabilization of α-Al_2_O_3_ through hydroxyl-assisted diffusion mechanisms, as reported in previous studies [18,23].

### 3.2. Surface Topography (Optical Profilometry)

**Figure 9:**
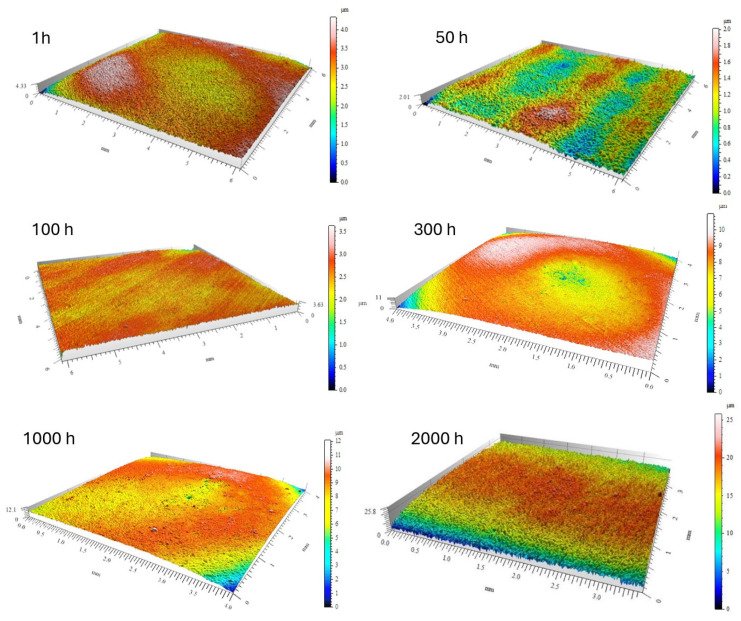
Three-dimensional image of the surface of the Fe40Al5Cr0.2TiB alloy oxidized in a steam environment at 700 °C for different times.

**Figure 10:**
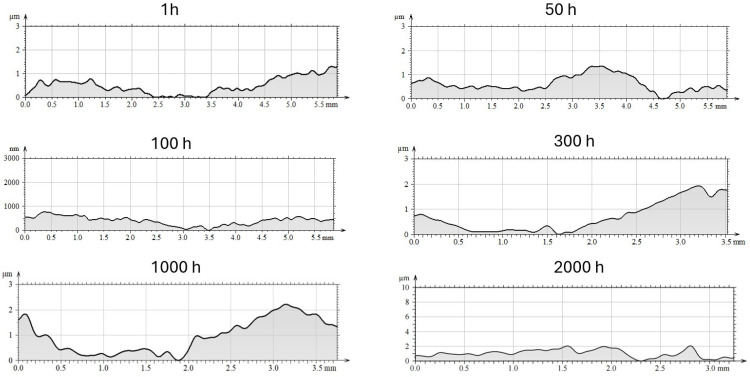
Waviness profiles on selected cross-sections of the sample surfaces after oxidation in a steam environment at 700 °C after different times.

The evolution of surface roughness parameters with oxidation time, determined by optical profilometry, is summarized in Table 2. The mean roughness (R_a_) and maximum height (R_z_) showed an initial slight decrease after 50–100 h of exposure (R_a_ ≈ 0.09 µm, Rz ≈ 0.57 µm), suggesting that fine-grained alumina layers partially smoothed the surface (Figure 9 and Figure 10). However, after 2000 h, both R_a_ and R_z_ increased markedly to 0.83 µm and 5.79 µm, respectively, reflecting the formation of coarse α-Al_2_O_3_ nodules and localized delamination zones.

The correlation between the increasing roughness and the observed SEM morphologies indicates that long-term oxidation in steam promotes thickening and sintering of the oxide scale, leading to the development of surface asperities and cracks [4,19,20]. These changes are consistent with models of alumina scale growth under stress accumulation and void formation beneath the oxide layer [10,26].

The increase in R_a_ and R_z_ parameters is therefore a quantitative signature of the morphological transition from a fine protective θ-Al_2_O_3_ whisker network to a coarse α-Al_2_O_3_ granular scale. The combination of SEM, EDS, and profilometry analyses clearly demonstrates that steam not only accelerates the θ → α transformation but also modifies the topography of the oxide, leading to increased roughness.

## 4. Analysis of the Results and Conclusions

The morphology of the oxide layer formed during steam oxidation, observed by scanning electron microscopy (SEM), at the initial stage of oxidation consists of flake-like regions. With increasing oxidation time, oxide structures in the form of needle-shaped whiskers growing from the substrate toward the oxidizing atmosphere were identified on the surface. Prolonged oxidation (2000 h) resulted in the formation of oxides with a granular morphology. Their nucleation and growth depend on local oxidation conditions, as indicated by their nonuniform and dispersed distribution over the surface. The whisker-shaped oxides, which crystallized during the early stages of oxidation, gradually transformed with increasing exposure time, disappearing and giving rise to more compact, flat crystallites.

The presence of aluminum oxide on the surface of the Fe40Al5Cr0.2TiB alloy in the form of fine needle-like structures was also reported in oxidation studies conducted by Nowak K. and Kupka M. [31], although this morphology appeared only during oxidation at a higher temperature of 800 °C for 200 h. At 900 °C, the needle-like oxide morphology was more pronounced. A similar needle-shaped oxide morphology was also observed in [32] during oxidation at 750 °C and 950 °C for 550 h. Comparable oxide morphologies have been reported not only for FeAl-based intermetallic alloys but also for NiAl alloys, which, like FeAl, crystallize in the B2 structure. In the case of NiAl, however, the needle-like aluminum oxide morphology was observed during oxidation at 1000 °C [33,34].

These observations indicate that both the morphology of corrosion products forming on FeAl-based intermetallic alloys and the kinetics of the oxidation process depend on the oxidation temperature and exposure time. All the above-mentioned researchers have noted that the needle-like oxide morphology is characteristic of the θ-Al_2_O_3_ phase. The morphology of oxides formed on the Fe40Al5Cr0.2TiB alloy obtained in this study can therefore be associated with the allotropic form of aluminum oxide present on the alloy surface. X-ray diffraction (XRD) analysis identified the presence of the α-Al_2_O_3_ phase. Importantly, the stable α-Al_2_O_3_ phase is generally recognized as a high-temperature modification of aluminum oxide, typically forming on the surface of oxidized materials at temperatures above 900 °C [31,35,36,37].

## Figures and Tables

**Table 1:** Chemical composition of Fe40Al5Cr0.2TiB alloy.

Compound	Fe	Al	Cr	Ti	B
% at.	54.80	40.10	4.86	0.18	0.06

**Table 2:** Summary of roughness parameters after oxidation.

Parameter	1 h	50 h	100 h	300 h	1000 h	2000 h
R_a_	0.119 µm	0.0847 µm	0.0921 µm	0.133 µm	0.175 µm	0.83 µm
R_z_	0.711 µm	0.486 µm	0.570 µm	0.785 µm	1.19 µm	5.79 µm

## Data Availability

The original contributions presented in this study are included in the article. Further inquiries can be directed to the corresponding authors.
